# Using Soluble Transferrin Receptor and Taking Inflammation into Account When Defining Serum Ferritin Cutoffs Improved the Diagnosis of Iron Deficiency in a Group of Canadian Preschool Inuit Children from Nunavik

**DOI:** 10.1155/2016/6430214

**Published:** 2016-06-13

**Authors:** Huguette Turgeon O'Brien, Rosanne Blanchet, Doris Gagné, Julie Lauzière, Carole Vézina

**Affiliations:** ^1^School of Nutrition, Laval University, 2425 rue de l'Agriculture, Québec City, QC, Canada G1V 0A6; ^2^Interdisciplinary School of Health Sciences, University of Ottawa, 35 University Private THN140, Ottawa, ON, Canada K1N 6N5; ^3^Nutrition DG, 1187 rue du Saint-Brieux, Québec City, QC, Canada G1Y 2B9; ^4^Department of Family Medicine and Emergency Medicine, University of Sherbrooke, 150 place Charles-Le Moyne, Longueuil, QC, Canada J4K 0A8; ^5^Inuulitsivik Health and Social Services Centre, Puvirnituq, QC, Canada J0M 1P0

## Abstract

The prevalence of iron depletion, iron deficient erythropoiesis (IDE), and iron deficiency anemia (IDA) was assessed in preschool Inuit children using soluble transferrin receptor (sTfR) and traditional indicators of iron status while disregarding or taking inflammation into account when defining SF cutoffs. Iron depletion was defined as follows: (1) SF < 15 *μ*g/L regardless of the C-reactive protein (CRP) level and (2) SF < 15 or <50 *μ*g/L with CRP ≤ 5 or >5 mg/L, respectively. IDE corresponded to iron depletion combined with total iron binding capacity > 72 *μ*mol/L and/or transferrin saturation < 16%. Iron depletion and IDE affected almost half of the children when accounting for inflammation, compared to one-third when the SF cutoff was defined regardless of CRP level (*P* < 0.0001). The prevalence of IDE adjusted for inflammation (45.1%) was very similar to the prevalence observed when sTfR was used as a sole marker of IDE (47.4%). The prevalence of anemia was 15%. The prevalence of IDA (IDE + hemoglobin < 110 g/L) was higher when accounting for than when disregarding inflammation (8.0% versus 6.2%, *P* = 0.083). Using sTfR and different SF cutoffs for children with versus without inflammation improved the diagnosis of iron depletion and IDE. Our results confirm that Inuit children are at particularly high risk for iron deficiency.

## 1. Introduction

Iron deficiency (ID) typically exists in three overlapping stages: depletion of storage iron, decreased iron transport within the body resulting in iron deficient erythropoiesis (IDE), and iron deficiency anemia (IDA) when IDE is combined with low hemoglobin. ID can have several adverse effects on children's health including impaired growth, poor cognitive and motor development, lethargy, and alterations of immune defense mechanisms [1].

Anemia from all causes affects almost 50% of preschool-aged children worldwide [2] and, according to the WHO, IDA is the most common type of anemia [3]. Although in industrialized countries the prevalence of ID among young children has been greatly reduced with the advent of food fortification, it continues to be an important public health problem among young Aboriginal children from Canada's remote north. There are only a few studies available on the prevalence of anemia and ID among preschool children. Recent data from a nationally representative sample of 3- to 5-year-old Canadian children revealed that 0.5% were anemic and 3.3% had depleted iron stores [4] compared with 16.8% and 18% for a group of Inuit children recruited at random from 16 Nunavut communities [5].

However, the impact of a concomitant inflammatory state which can increase serum ferritin (SF) levels in spite of depleted iron stores has received little attention in Inuit children. One approach to ensure a more accurate assessment of iron status is to measure an acute phase protein such as C-reactive protein or *α*1-acid glycoprotein and exclude persons with inflammation from the analysis [5, 6]. This approach can potentially underestimate the prevalence of ID [7] and can also be impracticable where the prevalence of inflammation is extensive [7], such as in Canadian Inuit children [8]. A higher cutoff value for SF, extending the normal 12–15 *μ*g/L limit to 30–50 *μ*g/L, has also been used in the presence of inflammation [9, 10]. More recently, the measurement of soluble transferrin receptor (sTfR), a transmembrane glycoprotein that transfers circulating iron into developing red blood cells [11] and is thought to be unaffected by inflammation [12, 13], has been proposed as a novel approach to diagnose ID in patients with concurrent inflammation [14]. Moreover, the combination of measurements of iron stores and of functional tissue iron as represented by sTfR/log ferritin (sTfR-ferritin index: sTfR-FI) is thought to have a higher diagnostic power than sTfR or SF alone in detecting ID with concomitant inflammation [14, 15].

To our knowledge, no studies have reported the impact of adjusting SF cutoffs to take into account the influence of inflammation or used sTfR and sTfR-FI in the diagnosis of ID among preschool Inuit children. The purpose of this study was to determine the prevalence of ID in a group of preschool Inuit children attending childcare centres in Nunavik using sTfR and traditional laboratory indicators of iron status while taking into account or disregarding the influence of inflammation on SF cutoffs.

## 2. Methods

### 2.1. Study Population and Data Collection

The study methods have been described in detail elsewhere [16]. Briefly, this prospective research project was part of a Nutrition Program implemented in childcare centres of Nunavik (northern Québec, Canada). The Nutrition Program, whose main purpose is to provide a balanced diet and reduce ID among Inuit preschool children, includes a four-week cycle menu (breakfast, lunch, and midafternoon snack) rich in absorbable iron. Information regarding total iron, bioavailable iron, and vitamin C content of the menu can be found elsewhere [16]. Data collection took place during the fall season between 2006 and 2010, and childcare centres located in ten of the fourteen Nunavik communities were visited. Parents or respondents of Inuit children aged 1–4 years and attending a childcare centre were invited to participate. Information on the study was provided individually, orally, or through a DVD available in Inuktitut, English, and French. Parents or respondents of participating children gave their written informed consent. This study was approved by the Research Ethics Board of the Centre Hospitalier de l'Université Laval du Centre Hospitalier Universitaire de Québec (CHUL-CHUQ), Québec (Canada). A total of 245 children were recruited between 2006 and 2010. The respondent for the child at the time of the interview was the biological mother (78.8%, *n* = 193) or father (2.8%, *n* = 7), the adoptive mother (15.5%; *n* = 38), the foster mother (2.4%, *n* = 6), or the foster father (0.4%, *n* = 1). Of the 245 children recruited for this study, a smaller number of participants had at least one laboratory indicator of iron status (*n* = 180) due to challenges related to drawing blood from young children [17]. Also, 162 children had a complete set of traditional laboratory indicators of iron status, whereas 149 had a sTfR measurement.

### 2.2. Blood Sampling and Laboratory Analyses

A nonfasting venipuncture blood sample was drawn into a vacutainer tube containing EDTA (BD 367863, Becton Dickinson and Co.) and into an iron free plastic tube. Blood samples were kept at room temperature, kept in an insulated container with ice packs, or refrigerated at 4°C for a maximum of 20 min prior to processing. For the determination of the complete blood count, whole blood aliquots of 0.5 mL (Sarstedt 2 mL tubes) were prepared, refrigerated, and sent to Inuulitsivik or Tulattavik hospitals located in Nunavik. Analyses were performed within 24 hours (exceptionally within 48 hours) using a Cell-Dyn 3200 haematology analyser (Abbott, Abbott Park, IL, USA).

The iron-free plastic tubes were centrifuged and the serum was isolated and aliquoted in Sarstedt 2 mL tubes. Within 3 h of collection, serum aliquots were frozen and stored at −18/−20°C. Frozen aliquots were kept in insulated containers with ice packs during transportation to laboratories in Québec City and Montreal. Serum iron (SI) and transferrin were measured on a Roche Modular system (Roche Diagnostics, Basel, Switzerland) using, respectively, the Ferrozine method without deproteinization and a standard particle-enhanced immunoturbidimetric assay. The coefficients of variation (intermediate precision) for transferrin and SI were, respectively, 1.4% and 1.8%. Percent transferrin saturation (TS) was calculated as follows: SI (*μ*mol/L)/TIBC (*μ*mol/L) × 100, where TIBC is total iron binding capacity, calculated as 25.1 × serum transferrin (g/L). SF was measured by electrochemiluminescence immunoassay using the Roche Modular E170 automated analyser (Roche Diagnostics, Basel, Switzerland). The precision was determined using Elecsys reagents in a protocol (EP5-A2) of the CLSI (Clinical and Laboratory Standards Institute) and corresponded to a coefficient of variation of 8.1%. High-sensitivity C-reactive protein (CRP) was determined by particle-enhanced immunonephelometry on a BN ProSpec nephelometer (Dade Behring, Siemens Healthcare Diagnostics, Liederbach, Germany). The total coefficient of variation for CRP was 5.8%. SI, transferrin, SF, and CRP were all measured at the Biochemistry Department, CHUL-CHUQ, Québec City. sTfR was performed with a nephelometric technique on a BN ProSpec System (Dade Behring, Siemens, Marburg, Germany) at the Immunology-Inflammation Laboratory, Notre-Dame Hospital, CHUM, Montreal. The total coefficient of variation of the sTfR method varied between 1.5 and 2.1% at concentrations between 0.14 and 4.4 mg/L. The sTfR-FI corresponded to the sTfR level divided by the log 10 of SF [18].

### 2.3. References Values and Iron Deficiency States

Anemia was defined as a Hb concentration < 110 g/L [19]. The cutoff values for red blood cell indices indicating, respectively, microcytosis and hypochromia were as follows: MCV < 72 fL [20] and MCHC (<320 g/L) [21]. SI < 9 *μ*mol/L [22, 23], TIBC > 72 *μ*mol/L [22, 23], and TS < 16% [14, 19, 23] are indicative of IDE. Elevated sTfR and sTfR-FI also reflect IDE [11, 24]. According to Vendt et al. every method used for measuring sTfR needs its own cutoff value [25]. The reference value recommended by the laboratory where our analyses were done was based on Raya et al. who showed that the 95th percentile of sTfR in children aged 3 to 10 years when measured on a Dade Behring nephelometer was 1.95 [26]. A sTfR-FI of 1.5 has been used as cutoff for ID in numerous studies [18, 27–29] and was taken in the current study. As summarized in Table 1, two methods based on traditional indicators of iron status (SF, TIBC, and TS) were used to define iron depletion, IDE, and IDA. In the first method, a SF cutoff < 15 *μ*g/L was used irrespective of the CRP level. In the second method, two different cutoffs were used for SF to take into account the absence (SF < 15 *μ*g/L) [30] or presence of inflammation (SF < 50 *μ*g/L) [6], which were defined as a CRP ≤ 5 mg/L or >5 mg/L, respectively [7, 31–33].

### 2.4. Data Analysis

Statistical analyses were conducted with SAS (version 9.2; SAS Institute Inc., Cary, NC, USA). Values are given as percentages (*n*), arithmetic means (SD), or medians with interquartile range (IQR: 25th–75th percentiles). The normality of distribution was revised both graphically and with the Shapiro-Wilk test. The spread of results was not normally distributed; thus, nonparametric statistical tests were used in this study. Because the Hb concentration was one of the most measured laboratory indicators among our participants, we used this parameter to compare the demographic and clinical characteristics of participants with (*n* = 180) or without (*n* = 65) an iron status indicator. The median test was used to compare the medians of breastfeeding duration, whereas the Wilcoxon rank-sum test and the chi-square test were used for continuous and categorical variables, respectively. We considered the following participant characteristics: age (months); sex (% male); coast of residence (% Hudson); breastfeeding status (% ever breastfed), breastfeeding duration (months); maternal age during pregnancy (years); supplement use during pregnancy (% yes); respondent's education (% secondary completed or more); and working status (% working). Breastfeeding duration was calculated only for breastfed children and was expressed as the median (IQR). For children who were still breastfed when the interview was conducted, it was equal to the child's age at the time of the interview. The McNemar test was used to compare differences in the prevalence of iron depletion, IDE, and IDA between the two methods (one SF cutoff versus two cutoffs). Spearman's correlation coefficients between different indicators of iron status were calculated. *P* value < 0.05 was considered a significant level.

## 3. Results

### 3.1. Population Characteristics

Demographic and clinical characteristics of participants with or without a Hb test are shown in Table 2. The mean age (SD) of children was 24.6 (9.8) months; about half of them were boys and lived on the Hudson coast. About seventy percent of these children had ever been breastfed (i.e., breastfed for some period). The median breastfeeding duration (IQR) in children who had been breastfed was 7.0 (2.5–15.0) months. The mean age (SD) of biological mothers during pregnancy was 23.7 (5.2) years. Eighty-one percent of mothers took a prenatal supplement during pregnancy. Almost two-thirds of mothers took a prenatal vitamin and mineral supplement (PVMS), while nearly a quarter of them took an iron supplement in addition to the PMMS (results not shown). About 38% of respondents had completed secondary schooling or more and 84% were working at the time of the interview. There were no significant differences in demographic and clinical characteristics of children who had at least one iron status indicator and those who had none (Table 2).

### 3.2. Iron Status

The mean (SD) Hb and SF concentrations were 120.2 (11.0) g/L and 24.5 (18.6) *μ*g/L, respectively (Table 3). The median (IQR) SF concentration was 21.0 (11.8–30.0) *μ*g/L. Among the other iron indicators, the mean sTfR-FI was 1.9 (1.8) and the median (IQR) was 1.5 (1.2–1.9). The prevalence of anemia from all causes (Hb < 110 g/L) was 15%. Microcytosis and hypochromia were found, respectively, in 22.8% and 27.7% of the study population. Low TS and increased TIBC, sTfR, and sTfR-FI, all indicative of IDE, were found in 81.8%, 74.1%, 47.4%, and 49.7% of children, respectively. Almost 25% of participants had a CRP concentration higher than the clinical cut-point for inflammation. Also, 32.9% of participants had depleted iron stores (SF < 15 *μ*g/L) irrespective of the CRP level.

As indicated in Table 4, almost half of the children had depleted iron stores when the absence or presence of inflammation was taken into account, whereas this proportion only reached one-third when SF was used irrespective of the CRP level (*P* < 0.0001). Similar results were observed for IDE and the difference between both methods was also statistically significant (*P* < 0.0001). With the exception of two participants, all the children with iron depletion also suffered from IDE. The prevalence of anemia from all causes was 15%. The prevalence of IDA was higher when two cutoffs were used for SF compared to using only one cutoff regardless of the CRP level (8.0% versus 6.2%, *P* = 0.083). It is noteworthy to mention that 3 of the remaining 13 children not classified as having IDA were very close to suffering from IDA in the absence of inflammation, except for their SF level that was slightly above the cutoff limit of <15 *μ*g/L (SF: 15 *μ*g/L, 15.3 *μ*g/L, and 17 *μ*g/L (results not shown)). Two other participants could not be classified as having IDA with coexistent inflammation due to the fact that their SF level was not <50 *μ*g/L, but rather 51 and 53 *μ*g/L (results not shown). In clinical practice, this would represent an additional 3% of participants classified as having IDA.

As shown in Table 5, there were significant correlations between Hb, MCV, and MCHC and all the other iron status indicators with the exception of Hb that was not significantly related to SF. Hb, MCV, and MCHC were negatively associated with TIBC, sTfR, and sTfR-FI. TIBC was negatively correlated with TS and SF but positively related to sTfR and sTfR-FI. TS was negatively related to sTfR and sTfR-FI but positively associated with SF. CRP was negatively correlated to Hb, MCV, MCHC, SI, and TS, whereas it was positively associated with sTfR and SF. Also of interest, sTfR and sTfR-FI were negatively correlated to SF.

## 4. Discussion

To our knowledge, this study is the first to determine the prevalence of iron deficiency in a group of young Inuit children using sTfR and sTfR-FI as well as a variety of conventional laboratory indicators of iron status while taking into account the absence or presence of inflammation.

### 4.1. Iron Depletion

Iron depletion, the initial stage of ID, is characterized by a low SF level. The mean SF concentration (SD) observed in our study was higher than the level reported in preschool Nunavut children (24.5 versus 19.1 *μ*g/L in Nunavut) [5]. This can be partially explained by a higher prevalence of inflammation in our study (CRP ≥ 8 mg/L: 18.8% (result not shown) versus 5.1% in Nunavut) which is associated with increased SF levels [34, 35]. The impact of CRP levels on SF values has also been observed in a group of Australian Aboriginal children from 6 to 60 months of age whose mean SF concentration was 56.3 *μ*g/L while 69.9% had a CRP level above 8 mg/L [36].

The prevalence of iron depletion irrespective of the CRP level affected one-third of our participants (Table 3). This is identical to the prevalence observed in older Inuit children (mean age: 11.3 years) from Nunavik [37]. However, we found a higher prevalence of iron depletion without inflammation than that found in a group of preschool Inuit children from Nunavut (SF < 12 *μ*g/L and CRP level < 8 mg/L: 29% (result not shown) versus 18% in Nunavut) [5]. This can partially be explained by the fact that preschoolers from Nunavik were twice as young as the ones from Nunavut (2.1 versus 4.4 years) and, thus, more susceptible to developing ID. Indeed, it has been reported in low income families that 1- to 2-year-old children were at higher risk of ID than 3- to 4-year-olds [38]. Moreover, a large database of 32,000 patients aged 12 to 59 months from Alberta (Canada) revealed that the peak age for IDA was 19 months [39].

In agreement with other studies [34, 35], we found that the use of two cutoffs for SF enabled us to identify significantly more children with depleted iron stores than when SF was used irrespective of the CRP level (Table 4). According to Wieringa et al., “using indicators of micronutrient status without considering the effects of the acute phase response results in a distorted estimate of micronutrient deficiencies, whose extent depends on the prevalence of infection in the population” [34]. We found that a quarter of our participants had an elevated CRP level confirming the high infection rate observed in many Canadian Inuit children [8]. However, the prevalence of inflammation observed in the present study was probably greater than revealed due to the fact that CRP detects acute inflammation while *α*1-acid glycoprotein, not measured in our participants, better detects chronic inflammation [7, 9, 35]. Considering that infections are more likely to occur in ID children [40], the exclusion of subjects with inflammation can reduce the prevalence of iron depletion aside from preventing the use of valuable data [34] making comparison with other studies more difficult.

### 4.2. Iron Deficient Erythropoiesis

When storage iron becomes depleted, the second stage of ID known as IDE develops. It is characterized by a decrease in iron being transported within the body resulting in low SI, increased TIBC, and reduced TS [23]. In agreement with other studies [41], we found that the proportions of children who were iron deficient based on these indicators used separately varied considerably (Table 3).

In the present study, elevated sTfR levels were found in almost half of the participants indicating that iron stores were low which was followed by an increase in sTfR. Indeed, during the process of iron depletion, sTfR concentration remains stable [15]. However, when iron stores are depleted, as indicated by a subnormal SF level, the concentration of sTfR increases [15]. Calculation of the sTfR-FI has been proposed to take advantage of the reciprocal relationship between SF and sTfR [42]. Yet, the proportion of abnormal values using the sTfR-FI and sTfR alone was similar in our study (49.7% versus 47.4%, Table 3) indicating that both indices were good indicators of IDE among our participants. Kamer et al. also reported in 6- to 36-month-old children that both sTfR and the sTfR-FI were good indicators of ID and could be useful in the differential diagnostics of anemia, especially in young children [43]. On the contrary, the log of sTfR-SF ratio has been described as a better criterion than either sTfR or sTfR-FI for defining the iron status of children, although the sTfR-SF ratio was not able to exclude IDA in the presence of inflammation [14]. Interestingly, Cook et al. used the sTfR-SF ratio to define an algorithm for estimating body iron in adult subjects [44]. However, the use of the sTfR-SF ratio to estimate body iron has certain limitations. Indeed, the extent to which this algorithm can be applied in school-aged and preschool-aged children is uncertain, whereas one of its main limitations is the influence of inflammation on SF levels independent of body iron stores [44]. Considering the high prevalence of inflammation found among our participants, the use of sTfR-SF ratio to assess the iron status based on the quantitative measurement of body iron would have been premature.

We also observed that the prevalence of IDE based on three traditional indicators of iron status in addition to CRP was similar to the proportion found when sTfR was used as a sole marker of IDE (45.1% (Table 4) versus 47.4% (Table 3)). When using traditional indicators of iron status, we raised the cutoff value for SF from 15 to 50 *μ*g/L with an elevated CRP level, whereas sTfR has been shown to be unaffected by concomitant infectious or inflammatory conditions [18, 45]. Even though sTfR assay is not available everywhere [46] (as is the case in Nunavik), it is set to become a useful diagnostic tool in clinical settings [11]. Until sTfR is more easily available, using different cutoffs for SF in the absence or presence of inflammation can significantly improve the diagnosis of IDE.

### 4.3. Iron Deficiency Anemia

In the present study, the prevalence of anemia from all causes (15%) (Table 3) was similar to that reported in Inuit preschool-aged children from Nunavut [5] but slightly more elevated than the one found among Québec Cree infants screened between 2002 and 2007 (12.5%) [47]. Nevertheless, before screening for anemia was initiated in 1995 in the Cree region of Québec, the prevalence of anemia was much higher reaching 31.7% [47].

In agreement with other studies [3], we found that IDA was the most common type of anemia among our participants. Indeed, there were 8% of children with IDA when using different cutoffs for children with versus without inflammation (Table 4), whereas an additional 3% of participants would have been classified as having IDA except for a borderline SF. Determining a cutoff limit for SF in the presence of inflammation is problematic and values between 10 and 100 *μ*g/L have been suggested to represent a diagnostic grey zone [48].

The prevalence of IDA observed in these preschool Inuit children from Nunavik (8%) was almost identical to the level reported in older children and adolescents also from Nunavik (8- to 10-year-olds) (8.7%) [37]. Although the determinants of anemia in children seem to vary according to the age bracket [49], the prevalence of IDA in Nunavik appears to remain stable throughout childhood and adolescence.

To our knowledge, there are currently no national statistics for the prevalence of IDA in Canadian children. However, a study carried out by Obaid in Edmonton (Alberta) (cited by Hartfield) and using a large database of more than 32,000 patients 12 to 59 months of age, between 2002 and 2008, indicated that the prevalence of IDA was 7.7% [39], which is almost identical to the rate observed in the current study. In USA, the prevalence of IDA in toddlers is estimated to be 1.2% [50], although higher prevalence has been observed in American children from high-risk population and low income families (3–6%) [38, 51]. Nevertheless, in the studies just mentioned above, children with an elevated CRP concentration (≥10 mg/L) were excluded from the analyses [38], or an acute phase protein such as CRP was neither measured [50, 51] nor reported [39]; thus the real prevalence of IDA might be higher than reported.

IDA is the most common cause of microcytosis and hypochromia [52, 53]. Microcytosis develops either ahead of or at the same time as any reduction in the Hb level [54]. A low MCV could indicate early ID that has not yet resulted in anemia [54]. This seems to be the case in our study as almost a quarter of the children had a low MCV (Table 3) while the prevalence of anemia was 15%. A decrease in MCH and MCHC, reflecting red cell hypochromia, can also be used to diagnose IDA. However, since hypochromia normally accompanies microcytosis, MCH [54] and MCHC are not more reliable than the MCV for detecting iron deficiency.

### 4.4. Correlation Coefficients between Iron Status Indicators

In our study, correlations were observed between traditional laboratory indicators of iron status and sTfR. As expected, there was a positive association between sTfR, sTfR-FI, and TIBC confirming results from previous studies which have shown that ID unquestionably leads to a significant rise of sTfR levels and TIBC [11, 22]. However, sTfR was negatively related to TS, SI, and MCV and, to a lower extent, to SF. The weakest correlation observed between SF and sTfR is not surprising since SF is a measure of iron stores whereas sTfR is a measure of tissue iron. The correlation found between sTfR and MCHC is not as strong as the one observed between sTfR and MCV. This is likely due to the fact that MCHC tends to be the last indicator to fall as ID worsens [55]. As reported by other authors, we observed a positive association between CRP and sTfR [9], as well as CRP and SF [9, 12, 32]. We also found a negative association between CRP and Hb [9, 12, 32], as well as CRP and SI [56].

## 5. Conclusion

The prevalence of anemia from all causes was 15%, thus near the upper limit defined by the WHO for mild anemia (5% to 19.9%) [3]. As expected, ID was the main cause of anemia among our participants. Moreover, accounting for the elevating effect of inflammation on SF significantly improved the diagnosis of iron depletion and IDE which affected almost half of the children compared to one-third when disregarding inflammation. Interestingly, taking inflammation into account when using traditional indicators of iron status resulted in a prevalence of IDE that was almost identical to the prevalence observed when sTfR, thought to be unaffected by inflammation [12, 13], was used as a sole marker of IDE. Although ID remains a public health concern in Canadian children, our results confirm that Aboriginal children are at particularly high risk [57]. However, the iron status of participating children could be better than that of Nunavimmiut children not enrolled in childcare since nonattendance to childcare has been identified as a risk factor for ID and IDA [57]. Strategies for preventing or reducing ID in preschool Inuit children from Nunavik are therefore needed.

## Figures and Tables

**Table 1:** Diagnostic criteria defining iron depletion, IDE, and IDA based on traditional indicators of iron status and CRP.

	Traditional indicators of iron status and CRP^*∗*^					
	Hb (g/L)	CRP (mg/L)	SF (*µ*g/L)	TIBC (*µ*mol/L)	and/or^†^	TS
SF cutoff < 15 *µ*g/L regardless of CRP level						
Iron depletion^†^	—	—	<15	—		—
IDE	—	—	<15	>72	or	<16
IDA	<110	—	<15	>72	or	<16

SF cutoffs < 15 or <50 *µ*g/L w/o or w/ inflammation, respectively^‡^						
Iron depletion w/o inflammation^§^	—	≤5	<15	—		—
Iron depletion w/ inflammation^§^	—	>5	<50	—		—
IDE w/o inflammation	—	≤5	<15	>72	or	<16
IDE w/ inflammation	—	>5	<50	>72	or	<16
IDA w/o inflammation	<110	≤5	<15	>72	or	<16
IDA w/ inflammation	<110	>5	<50	>72	or	<16

IDE: iron deficient erythropoiesis; IDA: iron deficiency anemia; CRP: C-reactive protein; Hb: hemoglobin; SF: serum ferritin; TIBC: total iron binding capacity; TS: transferrin saturation; w/o: without; w/: with.

^*∗*^Reference sources for the cutoffs were as follows: Hb [19]; CRP [7, 31–33]; SF [6, 30]; TIBC [22, 23]; TS [14, 19, 23].

^†^The choice “and/or” applies to the last two biochemical parameters.

^‡^SF cutoffs were adjusted for inflammation using the C-reactive protein (CRP): w/o inflammation: CRP < 5 mg/L; w/ inflammation: CRP ≥ 5 mg/L.

^§^Without or with concomitant IDE and IDA.

**Table 2:** Demographic and clinical characteristics of preschool Inuit children with or without a Hb test.

Characteristics	With a Hb test^*∗*^(*n*)	Without a Hb test^*∗*^(*n*)	*P* ^†^
Age (months)	24.6 (9.8) (180)	25.0 (9.5) (65)	0.64
Sex (% male)	52.2 (180)	52.3 (65)	0.99
Coast of residence (% Hudson)^‡^	52.8 (180)	55.4 (65)	0.72
Any breastfeeding			
Ever breastfed (% yes)	70.8 (178)	70.8 (65)	0.99
Breastfeeding duration (months)^§^	7.0 (2.5–15.0) (126)	6.2 (2.0–15.0) (46)	0.58
Maternal age during pregnancy (years)	23.7 (5.2) (163)	23.7 (5.7) (58)	0.74
Supplement use during pregnancy (% yes)	81.3 (155)	82.5 (57)	0.85
Respondent education level (secondary completed or more (%))^||^	37.8 (180)	44.6 (65)	0.43
Respondent working status (% working)^||^	83.9 (180)	81.5 (65)	0.66

Values are given as arithmetic means (±SD) or percentages unless otherwise specified.

^*∗*^180 participants had a Hb test, while 65 had none. Some characteristics, namely, breast feeding status, maternal age, and supplement use during pregnancy, were not available for all the children.

^†^Differences were assessed using a Wilcoxon rank-sum test for continuous variables, chi-square statistics for categorical variables, and the median two-sample test for breastfeeding duration.

^‡^Nunavik occupies a third of the province of Quebec (Canada) north of the 55th parallel and is bordered by the Ungava Bay (east), Hudson Strait (north), and Hudson Bay (west).

^§^Breastfeeding duration in breastfed children. Duration was equal to the child's age for children who were still breastfed at the time of the interview. Median (IQR: interquartile range).

^||^The respondent for the child at the time of the interview was the biological mother (78.8%, *n* = 193) or father (2.8%, *n* = 7), the adoptive mother (15.5%; *n* = 38), the foster mother (2.4%, *n* = 6), or the foster father (0.4%, *n* = 1).

**Table 3:** Prevalence of abnormal values for iron status indicators regardless of inflammation and C-reactive protein (CRP) in preschool Inuit children.

	Mean (SD)	Median (IQR)	Cutoff point^*∗*^	Prevalence of abnormal values % (*n*)
Hb (g/L)	120.2 (11.0)	120 (114–128)	<110	15.0 (27/180)
MCV (fL)	74.5 (5.7)	75.4 (72.3–77.9)	<72	22.8 (41/180)
MCHC (g/L)	330.0 (17.0)	329 (319–344)	<320	27.7 (49/177)
SI (*µ*mol/L)	8.7 (4.7)	8.0 (5.0–11.0)	<9	54.7 (93/170)
TIBC (*µ*mol/L)	81.3 (13.8)	80.6 (71.3–89.4)	>72	74.1 (126/170)
TS (%)	11.1 (6.5)	10.0 (6.3–14.0)	<16	81.8 (139/170)
SF (*µ*g/L)	24.5 (18.6)	21.0 (11.8–30.0)	<15	32.9 (56/170)
sTfR (mg/L)	2.1 (0.7)	1.9 (1.7–2.3)	>1.95	47.4 (73/154)
sTfR-FI	1.9 (1.8)	1.5 (1.2–1.9)	>1.5	49.7 (74/149)
CRP (mg/L)	5.9 (12.6)	0.9 (0.5–4.8)	>5	24.7 (42/170)

SD: standard deviation; IQR: interquartile range.

Hb: hemoglobin; MCV: mean corpuscular volume; MCHC: mean corpuscular hemoglobin concentration; SI: serum iron; TIBC: total iron binding capacity; TS: transferrin saturation; SF: serum ferritin; sTfR: soluble transferrin receptor; sTfR-FI: soluble transferrin receptor-ferritin index.

^*∗*^Reference sources for the cutoffs were as follows: Hb [19]; MCV [20]; MCHC [21]; SI and TIBC [22, 23]; TS [14, 19, 23]; SF [6, 30]; sTfR [26]; sTfR-FI [18, 27–29]; CRP [7, 31–33].

**Table 4:** Prevalence of iron depletion, IDE, and IDA in preschool Inuit children while disregarding or accounting for the influence of inflammation on SF cutoffs.

Iron status	*n*	Prevalence (%)	95% CI	*P* ^*∗*^
Iron depletion^†^				
SF < 15 *µ*g/L regardless of CRP level	53	32.7	25.4, 40.0	<0.0001
SF < 15 or <50 *µ*g/L w/o or w/ inflammation, respectively^‡^	75	46.3	38.5, 54.0	
IDE^§^				
SF < 15 *µ*g/L regardless of CRP level	51	31.5	24.2, 38.7	<0.0001
SF < 15 or <50 *µ*g/L w/o or w/ inflammation, respectively^‡^	73	45.1	37.3, 52.8	
IDA^§^				
SF < 15 *µ*g/L regardless of CRP level	10	6.2	2.4, 9.9	0.083
SF < 15 or <50 *µ*g/L w/o or w/ inflammation, respectively^‡^	13	8.0	3.8, 12.2	

IDE: iron deficient erythropoiesis; IDA: iron deficiency anemia; SF: serum ferritin; w/o: without, w/: with.

^*∗*^The McNemar test was used to compare differences in the prevalence of iron depletion, IDE, and IDA between the two methods in children with a complete set of traditional indicators of iron status (*n* = 162).

^†^Without (*n* = 2) or with concomitant IDE and, in some cases, IDA.

^‡^SF cutoffs were adjusted for inflammation using the C-reactive protein (CRP): w/o inflammation: CRP < 5** **mg/L; w/ inflammation: CRP ≥ 5** **mg/L.

^§^IDE and IDA as defined in Table 1.

**Table 5:** Spearman's correlation coefficients (*R*) between the different iron status indicators^*∗*^.

	Hb	MCV	MCHC	SI	TIBC	TS	sTfR	sTfR-FI	SF	CRP
Hb	1.0									
MCV	0.451^*∗*^	1.0								
MCHC	0.592^*∗*^	0.414^*∗*^	1.0							
SI	0.295^*∗*^	0.501^*∗*^	0.196^§^	1.0						
TIBC	−0.227^‡^	−0.450^*∗*^	−0.297^*∗*^	−0.123	1.0					
TS	0.352^*∗*^	0.598^*∗*^	0.280^†^	0.952^*∗*^	−0.390^*∗*^	1.0				
sTfR	−0.348^*∗*^	−0.650^*∗*^	−0.371^*∗*^	−0.547^*∗*^	0.499^*∗*^	−0.670^*∗*^	1.0			
sTfR-FI	−0.252^‡^	−0.552^*∗*^	−0.365^*∗*^	−0.294^†^	0.630^*∗*^	−0.473^*∗*^	0.782^*∗*^	1.0		
SF	0.098	0.310^*∗*^	0.205^‡^	−0.007	−0.537^*∗*^	0.158^§^	−0.340^*∗*^	−0.828^*∗*^	1.0	
CRP	−0.167^§^	−0.252^‡^	−0.173^§^	−0.625^*∗*^	−0.080	−0.545^*∗*^	0.268^†^	0.019	0.328^*∗*^	1.0

Abbreviations are as defined in Table 3.

^*∗*^With the exception of correlation coefficients involving sTfR and sTfR-FI for which the *n* varied between 141 and 149 participants and MCHC between 159 and 177, the *n* for all the other correlation coefficients varied between 162 and 180 participants.

*P* (*P* value):  ^*∗*^ *P* ≤ 0.0001; ^†^ *P* ≤ 0.001; ^‡^ *P* < 0.01; ^§^ *P* < 0.05.

## Additional Points

Certain limitations of our study need to be considered. First, data comparison between studies using sTfR is hampered by the lack of comparability of results across manufacturer assays [45, 58]. According to the WHO, there is an urgent need for a reference material with a certified concentration of transferrin receptor to standardize sTfR assays [30]. Another limitation is the fact that we only measured CRP which better detects acute inflammation as opposed to *α*1-acid glycoprotein which is a more sensitive indicator of chronic inflammation. Also, our participants have not been randomly selected in childcare facilities of Nunavik. Nevertheless, this study provides valuable insight concerning the iron status of these children considering that 245 participants have been recruited between 2006 and 2010 in 10 of the 14 Nunavik communities. Even though laboratory indicators of iron status were not available for the 245 participants, no significant differences were observed between demographic and clinical characteristics of participants with at least one laboratory indicator of iron status and those who had none.
